# Effect of Pre-Heating on Residual Stresses and Deformation in Laser-Based Directed Energy Deposition Repair: A Comparative Analysis

**DOI:** 10.3390/ma17102179

**Published:** 2024-05-07

**Authors:** Usman Tariq, Sung-Heng Wu, Muhammad Arif Mahmood, Michael M. Woodworth, Frank Liou

**Affiliations:** 1 Department of Mechanical and Aerospace Engineering, Missouri University of Science and Technology, Rolla, MO 65409, USA; 2 Intelligent Systems Center, Missouri University of Science and Technology, Rolla, MO 65409, USA; 3 The Boeing Company, 5301 Bolsa Ave. H021-F241, Huntington Beach, CA 92647, USA

**Keywords:** laser-directed energy deposition, trapezoidal shape repair, tool path generation, residual stresses, part distortion

## Abstract

Laser-directed energy deposition (DED), a metal additive manufacturing method, is renowned for its role in repairing parts, particularly when replacement costs are prohibitive. Ensuring that repaired parts avoid residual stresses and deformation is crucial for maintaining functional integrity. This study conducts experimental and numerical analyses on trapezoidal shape repairs, validating both the thermal and mechanical models with experimental results. Additionally, the study presents a methodology for creating a toolpath applicable to both the DED process and Abaqus CAE software. The findings indicate that employing a pre-heating strategy can reduce residual stresses by over 70% compared to no pre-heating. However, pre-heating may not substantially reduce final distortion. Notably, final distortion can be significantly mitigated by pre-heating and subsequently cooling to higher temperatures, thereby reducing the cooling rate. These insights contribute to optimizing DED repair processes for enhanced part functionality and longevity.

## 1. Introduction

Engineering components often endure harsh environmental conditions during prolonged operation, leading to wear, deformation, and cracks [1,2]. These defects can significantly impact the component’s geometry and performance. For instance, gas turbine engines used in aerospace undergo high-loading conditions and can face external debris as well [3,4]. Repairing damaged components using additive manufacturing (AM) is often more cost-effective than replacing them entirely, particularly for high-value parts [5,6]. Creating new components from scratch is typically expensive, and repairing is a preferable option for saving resources while ensuring functionality [7,8]. Repair involves restoring a damaged component to its initial operational state by introducing appropriate materials into the affected region until the original shape is reconstructed [9]. For metal AM, the process generally includes machining the damaged area to remove irregular surface defects and then introducing suitable materials through welding or additive manufacturing methods [10,11]. Laser-aided directed energy deposition (DED) has emerged as a promising technique for component repair [12]. DED utilizes a high-laser power to melt feedstock and deposit it in a layer-by-layer fashion onto the workpiece, forming fully dense parts with intricate geometries [13,14]. The laser creates a melt pool on the damaged part’s surface, where the feedstock is melted, cooled, and solidified to form deposits. Due to their full density and strong bond with the base component, these deposits exhibit excellent mechanical properties, including high tensile strength and fracture toughness. Further advantages of using DED also include precise additive material as it is controlled by numerically controlled machines or robotic arms, thus resulting in precise final geometry [15,16].

While utilizing the DED process for repair shows promise, there are several factors to consider before implementing this method. These aspects include examining the side wall angles at which the repair is conducted, assessing the final residual stress to determine if material strength is impacted and whether it remains operational, and finally, evaluating the deformation caused by residual stress, as a deformed part may not fit back into its original position [17].

One of the common defects observed in metallic components subjected to fatigue loading during service is cracking [18,19]. These cracks cannot simply be filled with additive feedstock material. Presently, a wide range of feedstock materials such as steel, Co-Cr, Al-Si-Mg, and Ni alloys have been used for fusion and fabrication processes [20,21,22]. Various studies have explored the use of dissimilar materials during the joining process to reduce defects [23,24]. It has been identified that micro- and nano-particles can also reduce the defects and increase the mechanical characteristics of the fabricated components significantly [25,26]. Another approach is to deposit the functionally grading feedstocks during the fusion process, yielding parts with elevated mechanical properties [27,28]. During repair, after removing the damaged area, the deposited wall angle plays a crucial role in determining the bond quality in the repaired component [29]. Oh et al. [30] investigated the effect of wall angles by creating trapezoidal grooves with varying lengths, widths, and depths for stainless steel 316 repair. They observed that lower-angle walls resulted in better bonding and superior material properties, such as tensile strength, compared to higher wall angles. Similarly, Li et al. [1] conducted experimental and numerical analyses comparing V-grooves and rectangular grooves for residual stress and other mechanical properties. They found that a V-groove with a 45° angle not only facilitated good bonding between the repair and original part but also provided comparable tensile strength to the original component. In contrast, the rectangular groove with a 90° wall angle led to porosity due to insufficient fusion, hindering proper melt pool formation. Pinkerton et al. [31] investigated repair experiments on inclined surfaces produced through machining, followed by metal deposition on H13 tool steel. Their findings suggested that achieving proper metallurgical bonding on inclined surfaces is uncertain. Furthermore, Zhang et al. [32] conducted experimental studies on tool steel, depositing materials using the DED method on surfaces inclined at angles of 45°, 75°, and 90°. They concluded that perpendicular walls failed to establish metallurgical bonding, resulting in defects, whereas other angles showed no such issues. Paul et al. [33] conducted an experiment on WC-CO and low-carbon steel and were able to show good bonding at the interface with a high value of hardness. Zhang et al. [3,34] performed several experiments on damaged compressed blades and various other moving components using the DED process. It was concluded that side angles play an important role in good metallurgical bonding and, if not properly considered, may lead to defects like lack of fusion and low strength.

Another critical aspect for achieving effective repair on damaged components is to create a tool path that ensures proper volume filling for the repair, also known as the reconstruction of the repair volume. For instance, Wilson et al. [35] utilized the laser metal deposition method to repair damaged turbine blades with the aid of a toolpath algorithm, subsequently validating its tensile strength compared to nominal material. While creating a toolpath for simple geometries may be relatively straightforward and can be carried out manually [36], it becomes significantly more challenging when dealing with multiple components. Therefore, there is a strong need to develop a toolpath generator that ensures proper reconstruction of the repair volume and possesses the capability to be automated, accommodating various input file systems. This necessity elucidates one of the objectives of this study. After conducting the literature review as presented earlier, it is evident that most of the research has focused on the relationship between wall angle and metallurgical bonding, along with other material properties such as tensile strength and hardness. Moreover, it was noted that only a few studies have investigated residual stress formation in the repaired part [37]. The stresses can be evaluated using experimental, modeling, and machine learning-based approaches. These techniques have been widely adapted to a range of manufacturing processes [38,39,40]. The prime advantage of modeling is to reduce the experimental cost by replicating the real experimentation in a virtual environment [41]. Thus, yielding process–structure–property–performance relationships between inputs and outputs [42,43]. Within modeling, two approaches have been commonly explored, including finite element (FE) and analytical analyses [44,45]. FE analysis is resource-consuming due to the iterative nature involved during model solution while analytical analysis does not involve an iterative solution technique, resulting in less resource utilization compared to the FE simulation technique [46,47].

To the best of the authors’ knowledge, no studies were found that analyzed the final geometric dimensions in terms of deformation, which is essential to verify whether the repaired part can fit back into the original component. It is crucial to examine dimensional deviations. This study is based on three novel objectives, including tool path generation for a trapezoidal geometry that involves applying basic trigonometric principles, ensuring an elegant solution. The resulting code can be employed for an in-house-built DED machine and the Abaqus CAE AM Modeler plug-in. This method utilizes the event series approach to generate the tool path code. The calculation of residual stresses is performed using numerical methods, focusing on those formed during the DED process. Subsequently, a comparison is made between the final deformation observed in the experimental part and the deformation predicted by the 3D model, as well as to investigate the effect of pre-heating at various initial temperatures and different cooling rates on residual stress, particularly on final deformation.

## 2. Methodology and Materials

The methodology and materials employed in this study are elaborated in this section. Initially, the damaged area was assumed to be removed in a Trapezoidal shape on a rectangular substrate. Subsequently, the generation of the tool path, experimental study, and numerical analysis are discussed in detail below.

### 2.1. Tool Path Generation

**Figure 1:**
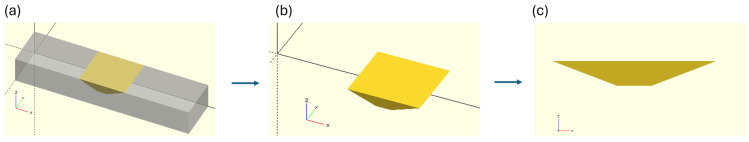
(**a**) Part with expected repair, (**b**) repair volume, and (**c**) front view of repair volume.

In reconstructing the part, it is important to calculate the repair volume to devise the tool path for repair, as this process accurately defines the geometry needed in the repair area. Thus, creating a CAD model of the part to be repaired, along with the repair volume, is crucial in determining how to generate an appropriate toolpath for the repair process. In this research, the gray color part with a trapezoidal groove is the part that needs to be repaired, as shown in Figure 1a. Figure 1b,c show the repair volume in 3D and front views, respectively. 

**Figure 2:**
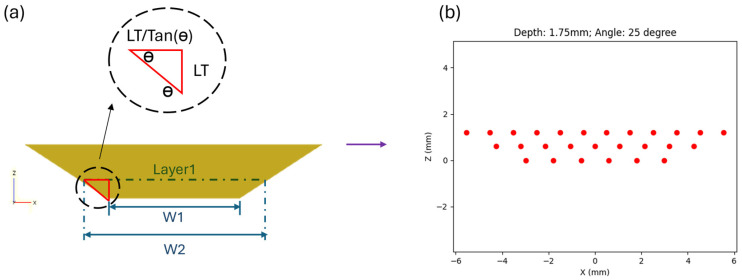
(**a**) Correspondence between geometric parameters and (**b**) repairing tool path with 1.75 mm depth and 25° geometry.

The repairing tool path essentially varies with geometric parameters, such as groove’s angle (*θ*), groove’s depth (*D*), layer thickness (*LT*), and process parameters, including laser beam size (*BS*) and hatching space (*HS*). This research shows how to generate the tool path corresponding to different groove geometries. Assuming that the *LT* is properly controlled and consistent, the tool path of each track is generated by first calculating the width of each layer using trigonometric functions, as demonstrated in Equation (1), where *W* denotes the layer width and *n* denotes the layer number. Secondly, the distribution of points along the *x*-axis, denoted by *X*_(*n*,*i*)_, is calculated to ensure uniform spacing within the designated width. This distribution is essential for generating an evenly spaced grid or pattern across a given layer (*n*) and index of point (*i*), as shown in Equation (2). Figure 2a demonstrates the schematic of the calculating concept, and Figure 2b demonstrates the result of tool path generation from the front view.

(1) $$W_{n}=W_{1}+\left(n-1\right)\times \left(\frac{2\times LT}{\tan\left(\theta \right)}\right).$$

(2) $$X_{\left(n,i\right)}=-X_{\left(n,i\right)}=-0.5\times W_{n}+i\times \left(\frac{W_{n}}{BS\times \left(1-HS\right)}\right);\;i\;\in \left\{0,1,2,\ldots ,\left[BS\times \left(1-HS\right)\right]\right\}.$$

To cope with various trapezoidal grooves, the tool path generator has been developed in this research. The groove’s angle, depth, laser beam size, hatching space, and layer thickness are considered in the tool path generator, so it can deal with the repairing process with different geometries and process parameters. 

### 2.2. Experimental Setup

**Figure 3:**
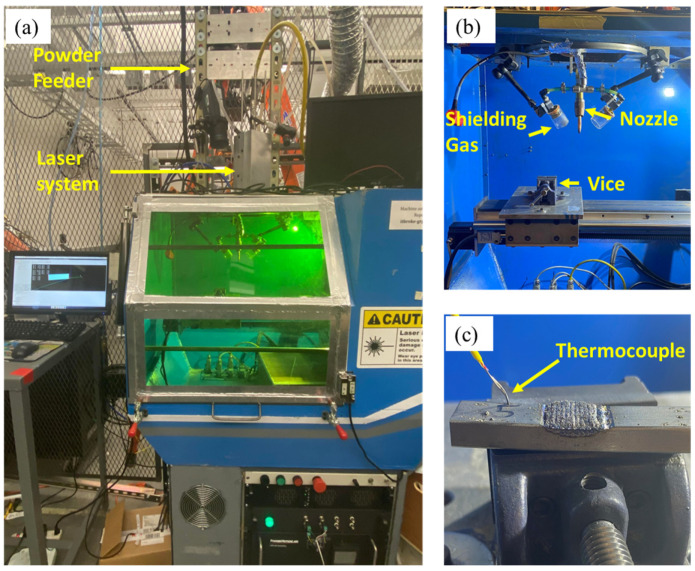
(**a**) In-house-developed DED system, (**b**) shielding gas and powder system, and (**c**) repaired part along with thermocouple.

The study utilized an in-house-developed DED, located in Missouri University of Science and Technology, Rolla, MO, USA, equipment featuring a neodymium-doped yttrium aluminum garnet optical laser system generating 1 kW of power. Figure 3a provides an overview of the DED machine system. Figure 3b showcases the shielding gas system equipped with an off-axis powder feeding nozzle, alongside a substrate holder capable of 3D movement. Figure 3c illustrates the specimen utilized in the study, accompanied by K-type thermocouples employed to extract temperature data for validating a numerical model. One thermocouple was affixed to the substrate via soldering to extract temperature measurements at intervals of 0.01 s.

**Figure 4:**
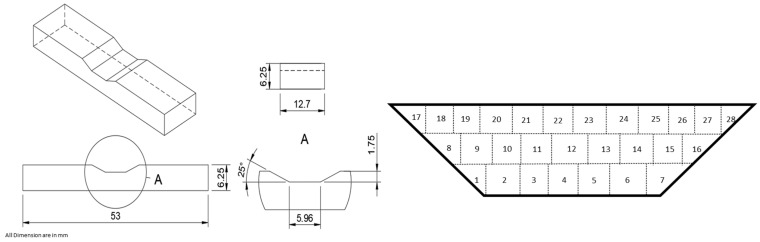
Dimension of the part to be repaired and scan pattern; all dimensions are in mm.

For the fabrication of the repaired trapezoidal geometry, the powder feed rate was set at 2.50 g/min, the Argon shielding gas pressure was fixed at 40.0 psi, and a laser beam with a 2.20 mm spot size, exhibiting a Gaussian heat distribution, was selected. A repaired structure comprising three layers was printed with specific printing parameters. These parameters included a laser power of 500.0 W and a laser scanning speed of 200.0 mm/min. Notably, the laser was turned off during the transition from the end of one track to the beginning of the next, ensuring precise and controlled printing. This approach facilitated the creation of a robust and accurate structure, meeting the desired specifications and requirements for the repair process. The detailed dimensions of the part to be repaired along with the scan pattern in trapezoidal geometry are shown in Figure 4.

### 2.3. Numerical Analysis

In this investigation, we have devised and implemented a 3D, sequentially coupled thermo-mechanical model employing the ABAQUS finite element (FE) analysis software with an AM plug-in. The aim is to replicate the transient temperature distribution and residual stresses occurring during the DED process of Ti6Al4V. The numerical simulation comprised two principal stages. Initially, a transient thermal analysis is executed to describe the thermal evolution across the entire workpiece. Subsequently, a mechanical analysis was carried out to determine the residual stress and deformation of the workpiece, utilizing the temperature field from the preceding step. The number of elements and nodes used in the simulation were 258,762 and 137,834 nodes, respectively. All computational simulations were conducted on a computer featuring an Intel(R) Xeon(R) W-2295 CPU clocked at 3.00 GHz, boasting 18 cores and 128 GB of RAM operating at 2934 GHz. The deposition time step was 133 s followed by 1 h of room cooling. For the thermal model, the computational time was 280 min, and for the mechanical model, it was around 391 min.

#### 2.3.1. Thermal Model

During the DED process, the stress and deformation within a structure predominantly hinge on the temperature field, with minimal influence from the stress and deformation field. Therefore, a heat transfer analysis that is decoupled from the mechanical effects is considered appropriate. For the thermal analysis, we utilized DC3D6. The transient temperature field, represented as *T* (*x*, *y*, *z*, *t*), encompassing the entire domain, was acquired by solving the three-dimensional heat conduction equation (Equation (3)) within the substrate while incorporating appropriate initial and boundary conditions [48,49,50,51]:

(3) $$\frac{𝜕}{𝜕x}\left(k\frac{𝜕T}{𝜕x}\right)+\frac{𝜕}{𝜕y}\left(k\frac{𝜕T}{𝜕y}\right)+\frac{𝜕}{𝜕z}\left(k\frac{𝜕T}{𝜕z}\right)+Q_{f/r}=\rho C_{p}\frac{𝜕T}{𝜕t}$$

where *k* represents the material’s thermal conductivity, *ρ* denotes the material’s density, *C_P_* stands for the material’s specific heat, and *Q_f_*_/*r*_ indicates the volumetric heat flux. Newton’s law of cooling, which calculates heat loss due to convection, is expressed as shown in Equation (4) [48,49,50,51]:

(4) $$q_{c}=h_{c}\left(T-T_{env}\right)$$

Here, *q_c_* represents the heat loss due to convection, *h_c_* stands for the convection heat coefficient and its value was 30.0 (W/m^2^. K), *T* denotes the surface temperature, and *T_env_* refers to the environmental temperature or initial temperature taken as 25.0 °C. Heat loss due to radiation is calculated using Stefan Boltzmann’s law, as shown in Equation (5) [48,49,50,51]:

(5) $$q_{r}=\varepsilon \sigma \;\left(T^{4}-T_{env}^{4}\right)$$

where *q_r_* denotes the heat loss due to radiation, *ε* represents the emissivity (0.8), and *σ* signifies the Stefan–Boltzmann constant (=5.67 × 10^−8^ W/m^2^. K^4^).

Goldak’s double ellipsoidal heat source was used in this simulation; its volumetric heat distribution is shown in Equations (6)–(8), and relevant parameters are presented in Table 1 [51].

(6) $$Q_{f/r}=\frac{6\sqrt{3}F_{f/r}q}{abc_{f/r}\pi \sqrt{\pi }}\;e^{-\left(\frac{3x^{2}}{c_{f/r}^{2}}\right)}e^{-\left(\frac{3y^{2}}{a^{2}}\right)}e^{-\left(\frac{3z^{2}}{b^{2}}\right)}\;$$

(7) $$Q=Q_{f}\;when\;x\geq 0$$

(8) $$Q=Q_{r}\;when\;x<0$$

where *q* is the power of the laser in Watts; *f* and *r* represent the front and rear parts of heat distribution; *F* is the fraction factor; *c*, *a*, and *b* are the width, breadth, and depth of the laser source in the relevant direction of *x*, *y*, and *z* respectively. Laser absorption or efficiency was taken at 0.38.

#### 2.3.2. Mechanical Model

The mechanical modeling, constituting the second step of numerical modeling in the DED process, can be computed using stress equilibrium, as expressed in Equation (9) [48,49,50,51]. For the mechanical analysis, we utilized C3D6, a node linear triangular mesh-type prism.

(9) 𝛻• σ+b=0

where 𝛻 • represents the divergence operator, *σ* denotes the stress tensor or Cauchy tensor, and *b* represents body forces.

All strains can be categorized into two main parts: (a) strains resulting from mechanical forces, and (b) strains induced by thermal loads, as mentioned in Equation (10) [48,49,50,51]. These strains can be further classified into five distinct types: strain due to elastic behavior, plastic deformation, thermal effects, phase transformation, and transformation of plasticity, as compiled in Equation (11) [48,49,50,51]. For the current analysis, only strains attributed to elastic, plastic, and thermal effects have been considered, as expressed in Equation (12) [48,49,50,51].

In Equation (10), *ε_ij_* represents the total strain, *ε_ij_^M^* denotes the strain resulting from the mechanical forces, and *ε_ij_^T^* signifies the strain due to thermal loads. In Equation (11), *ε_ij_^E^* represents the elastic strain, *ε_ij_^P^* denotes the strain due to plastic deformation, *ε_ij_*^∆*V*^ signifies the strain due to volumetric changes, and *ε_ij_^TRP^* represents the strain due to phase transformation.

(10) $$\varepsilon_{ij}=\varepsilon_{ij}^{M}+\varepsilon_{ij}^{T}.$$

(11) $$\varepsilon_{ij}=\varepsilon_{ij}^{E}+\varepsilon_{ij}^{P}+\varepsilon_{ij}^{T}+\varepsilon_{ij}^{∆V}+\varepsilon_{ij}^{TRP}$$

(12) $$\varepsilon_{ij}=\varepsilon_{ij}^{E}+\varepsilon_{ij}^{P}+\varepsilon_{ij}^{T}$$

The mechanical constitutive law employed in the current simulation is defined in Equation (13) [48,49,50,51]:

(13) $$\sigma =C\;:\;\varepsilon^{E}$$

where *C* represents the fourth-order elastic stiffness tensor.

### 2.4. Material Applied during Analyses

In this investigation, Titanium alloy (Ti6Al4V) was selected due to its numerous advantages in AM [52]. It boasts an exceptional strength-to-weight ratio, rendering it well-suited for applications requiring lightweight yet durable components, especially in aerospace [53]. Ti6Al4V demonstrates outstanding corrosion resistance, making it viable for use in challenging environments [54]. Its biocompatibility ensures suitability for medical and dental implants, ensuring compatibility with the human body. Moreover, Ti6Al4V’s compatibility with AM facilitates the fabrication of intricate geometries, rendering it a valuable material for the aerospace and automotive industries [12]. 

For FE analysis (FEA), accurate temperature-dependent properties are crucial to precisely model thermal and mechanical behavior [13]. In this study, Ti6Al4V’s temperature-dependent mechanical properties, as outlined in Table 2, were utilized to conduct the FEA analysis [49].

## 3. Results and Discussion

### 3.1. Thermal Validation and Results

**Figure 5:**
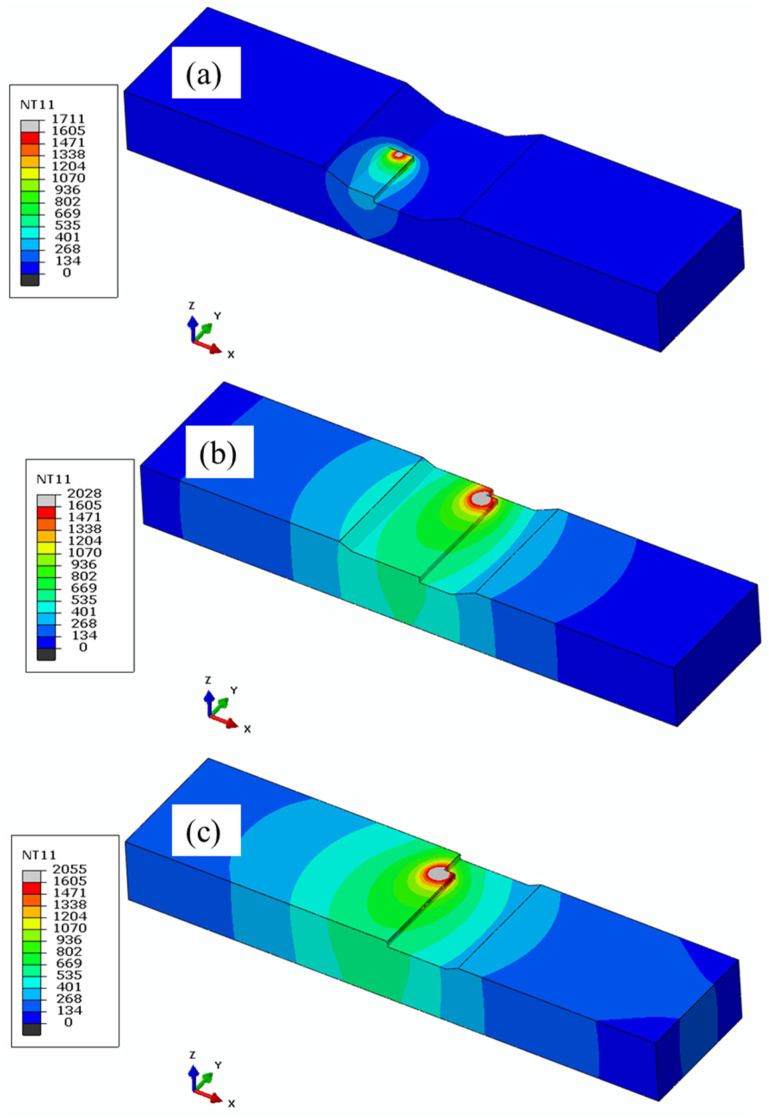
Temperature (°C) evolution during DED repair: (**a**) first layer, (**b**) second layer, and (**c**) third layer.

Figure 5 illustrates the evolution and temperature distribution in °C during the DED repair process of a trapezoidal defect shape. To ensure proper bonding, the first track of the first layer (Figure 5a) is analyzed, revealing a maximum temperature of 1711 °C, consistently above the melting temperature of 1605 °C throughout the track, indicating successful bonding. In Figure 5b, the 18th track on the second layer reached a maximum temperature of 2028 °C, with occasional spikes reaching up to 2214 °C. A similar trend was observed in the third layer, with temperatures reaching 2055 °C on the 21st track and spiking to 2388 °C, as shown in Figure 5c. This increasing temperature trend is attributed to heat accumulation during the laser deposition process, leading to subsequent temperature rises.

**Figure 6:**
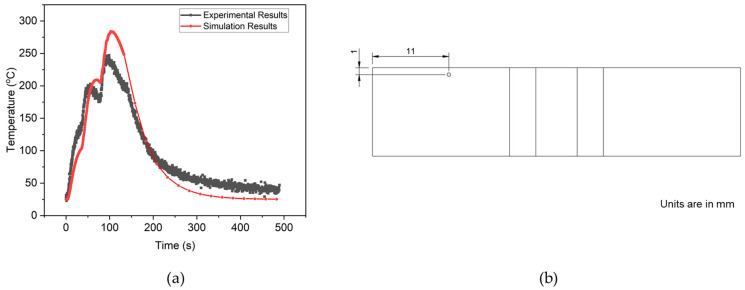
A comparison of (**a**) thermal history for both experimental and simulation results, and (**b**) the location of the thermocouple is shown from the top view.

In Figure 6a, the predicted simulation results, represented by nodal temperature values extracted at intervals of 0.5 s, are compared with the experimental analysis. The schematic of obtaining the experimental data via thermocouples is illustrated in Figure 6b, indicating their respective locations. Remarkably, both curves exhibit a closely matching trend with minimal deviation, demonstrating strong agreement. Notably, three distinct peaks are discernible on both curves, corresponding to the deposition of each layer. Following the first peak, the temperature curve sustains without significant cooling. However, a rapid cooling phase is observed after the second peak, attributed to higher temperature differentials. The cooling rate at the end of the third layer or deposition also aligns with experimental and simulation results.

### 3.2. Residual Stress Evolution and Final Deformation Validation

**Figure 7:**
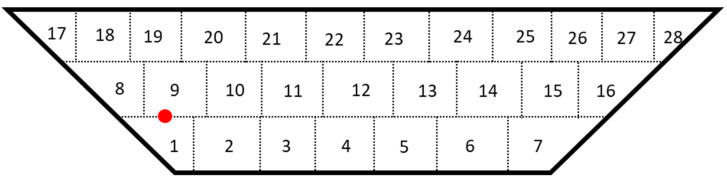
Scan patterns and location of point “A” on track 1.

**Figure 8:**
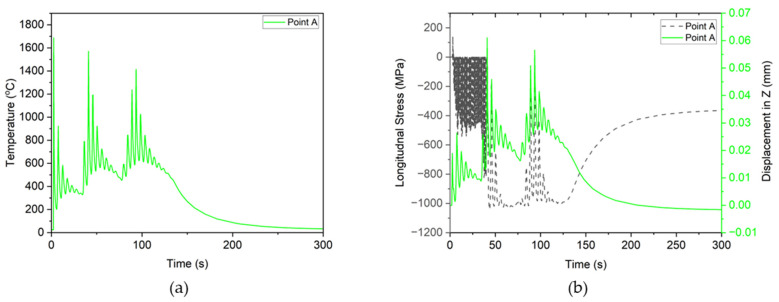
(**a**) Thermal cycling loads on point A, and (**b**) deformation and longitudinal stresses of point A.

To analyze the evolution of stress formation and its relationship with corresponding deformation in the *z*-direction, a nodal point was selected at the mid-length of track 1 as depicted in Figure 7. At point “A” (highlighted with red), a notable temperature spike occurs, peaking around 1790 °C, with subsequent decreases as the laser moves away, only to spike again upon return for the second and third layers. Initially, during the first temperature spike, longitudinal stresses were tensile due to material expansion during melting, resulting in positive deformation [55]. Subsequent spikes in the first layer led to fluctuating deformation due to extreme temperature variations. Following the initial tensile stress spike, the material cooled and began to shrink, resulting in the formation of compressive stresses in the longitudinal direction. As point “A” experienced temperature increases in subsequent layers, deformation and compressive stresses increased until deposition ceased, and the cooling process commenced. At this stage, the material reached equilibrium with a slight negative deformation of 0.00163 mm and compressive stresses of 363.8 MPa, as shown in Figure 8b.

**Figure 9:**
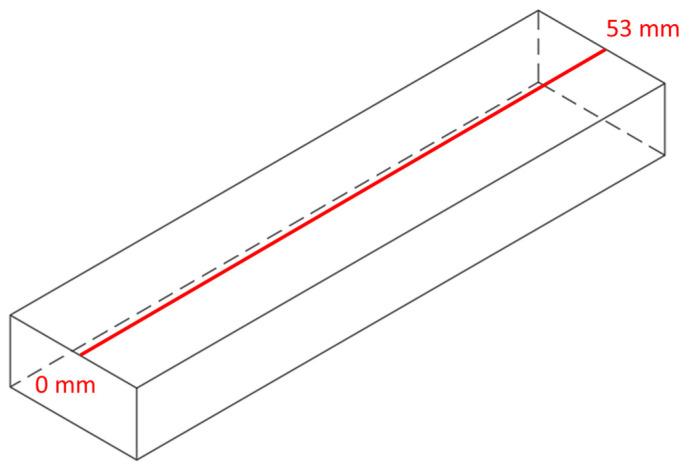
Values of temperature, residual stresses (longitudinal and transverse), and deflection in the z-direction were collected along the specified line.

To further investigate the evolution of longitudinal (S22) and transverse (S11) stresses, along with temperature and deflection, a straight line was chosen on the repaired part, as depicted in Figure 9. Nodal values were extracted at various time intervals after deposition and compared with the deflection in the *z*-direction, which was also designated as U3. U3 was selected for two reasons: it exhibited the highest deflection in this direction and was later validated through experimentation.

**Figure 10:**
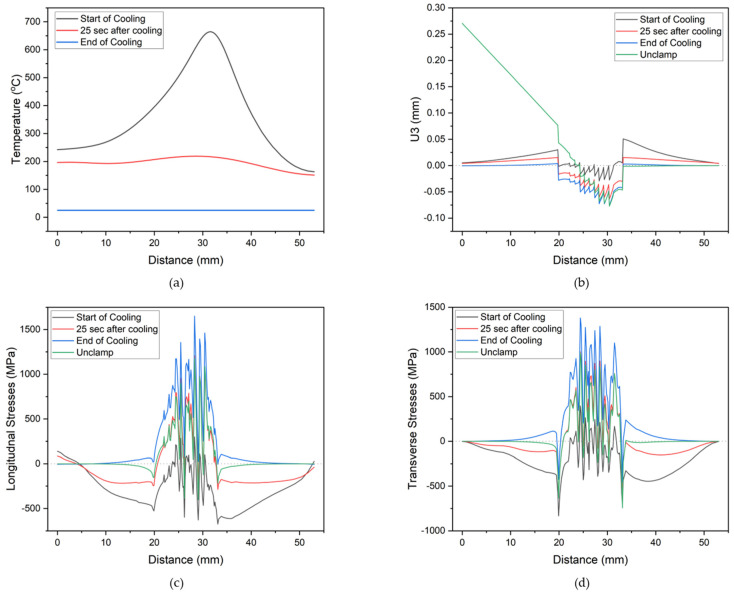
Values were collected along the specified line for (**a**) temperature, (**b**) deformation in the z-direction, (**c**) longitudinal stresses, and (**d**) transverse stresses.

Figure 10a depicts the temperature profile along the line at different times during the simulation, including the start of cooling or the end of the deposit, 25 s after cooling, and the end of cooling. It is evident that both ends gradually approach equilibrium conditions by the end of the simulation, reaching room temperature. A higher cooling rate is noticeable on the right side of the part, as this portion was clamped to the vice during deposition, thus facilitating more efficient heat transfer compared to air cooling.

Figure 10c,d illustrate the evolution of longitudinal and transverse stresses, respectively. Initially, both types of stresses exhibit similar behavior: at the start of cooling, the substrate or original part displays compressive stresses, while the repaired area experiences extreme fluctuations between compressive and tensile stresses, albeit mostly remaining in a compressive state. However, as the cooling progresses, these compressive stresses gradually transition into tensile stresses, as depicted in the figures following 25 s of cooling. This trend intensifies as the part cools down to room temperature, resulting in maximum tensile stresses peaking at 1379.0 MPa and 1651.0 MPa for transverse and longitudinal stresses, respectively. Upon unclamping the part, residual stresses are released, causing both transverse and longitudinal stresses to decrease to 987 MPa and 1175 MPa, respectively. Additionally, it is evident that since most of the compressive stresses are concentrated on the substrate and tensile stresses on the repaired part, the part tends to buckle inward.

Figure 10b illustrates the relevant U3 as the residual stresses evolve. At the start of cooling, it is observed that both ends exhibit zero deflection, but as we move toward the center, the deflection begins to increase in the positive direction, reaching its peak at the center before transitioning into the negative direction. This trend persists throughout the cooling process until 25 s and at the end of cooling. It is worth noting that when the repaired part is unclamped, the deflection measurement is adjusted such that the right end is placed downward, and maximum deflection is observed at the left side.

**Figure 11:**
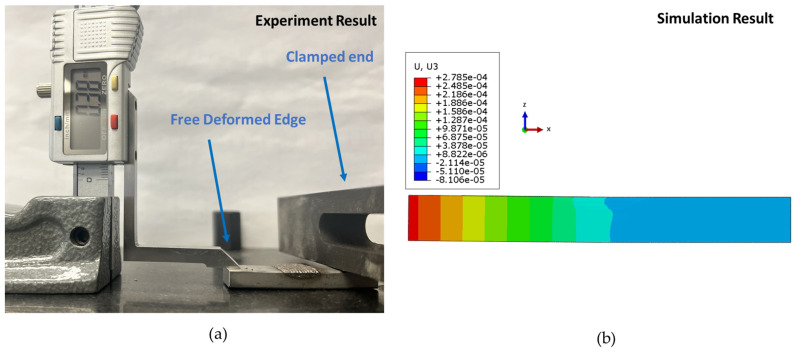
Measurement of deformation for final repaired part during (**a**) experiment (mm) and (**b**) simulation (m).

The observed trend suggests that with compressive stresses on the inside and tensile stresses on the outside of the repair, the final shape of the repaired part may deform inward. To verify this, a repaired part was clamped on one end, and deformation was measured using a height gauge, zeroing it at a length equal to the thickness of the substrate to measure only the deformation. These measurements were then compared to simulation results for validation purposes, with appropriate boundary conditions applied, as depicted in Figure 11a,b, respectively. The final deformation in the simulation was 0.278 mm, while for the experimental part, it was 0.38 mm. The difference in deformation falls within an acceptable range, validating the mechanical model. This error can be attributed to complex boundary conditions, such as the clamping of the specimen, as well as other physical conditions such as the Marangoni effect or varying radiation and convection coefficients, which were neglected in the model.

**Figure 12:**
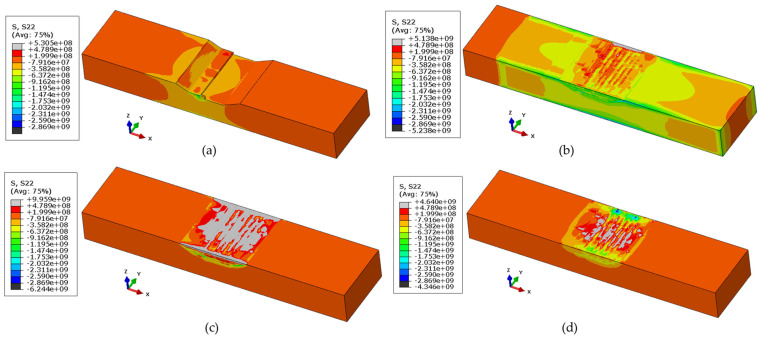
Evolution of longitudinal stresses (units in Pascal) while repairing (**a**) at the start of deposition, (**b**) at the end of the deposition, (**c**) at the end of the cooling period, and (**d**) after unclamping.

As the evolution of transverse and residual stresses follow the same pattern but in different directions, only the evolution of longitudinal stresses is depicted in Figure 12. Figure 12a illustrates the development of longitudinal stresses after the completion of the first track. At the end of the first track, when the laser is active, stresses are tensile. However, as the laser moves away, or at the start of the first track, these stresses begin to convert into compressive stresses. As deposition progresses and reconstructs the remaining groove, high compressive stresses can be observed in the lower layers and tensile stresses in the upper layer, as shown in Figure 12b. Additionally, compressive stresses can be observed at the sides of the rectangle due to reaction forces from the clamp, as the rectangle attempts to expand, resulting in compressive stresses on the part. At the end of the cooling and proper solidification, stresses appear to accumulate at the deposit or heat-affected zone, as shown in Figure 12c,d, which illustrate the stress condition once the part is unclamped, thus relieving some of the stresses as the part distorts.

**Figure 13:**
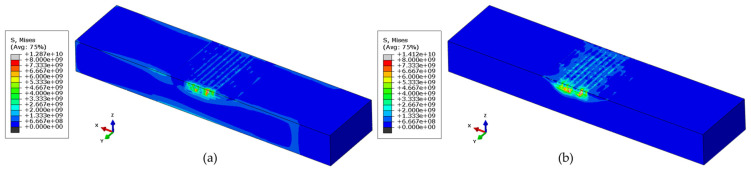
Von-mises stress (units in Pascal) criteria (**a**) at the end of the deposit and (**b**) after unclamping the repaired part.

A similar trend in von Mises stress criteria is evident, as depicted in Figure 13. At the end of the deposition and before cooling, von Mises stresses are dispersed throughout the part, as shown in Figure 13a. However, upon cooling down and unclamping from the vice, high stresses appear to accumulate at the deposit, as shown in Figure 13b.

### 3.3. Effect of Pre-Heat on Residual Stress and Final Deformation

From the analysis of residual stresses and final deformation of the part, it is evident that the part may not fit back properly to make it functional. Therefore, it is essential to explore methods to improve deformation and residual stresses. Various cases are suggested in this study. Case 1 serves as the baseline study, discussed previously. The substrate was pre-heated to 300 °C, 500 °C, and 700 °C and then cooled down to room temperature, forming Cases 2, 3, and 4, respectively. It is evident from various studies that the cooling rate affects the formation of final residual stresses and deformation. Therefore, two cases of varying cooling rates are also included in this section: (a) pre-heating the substrate to 700 °C and cooling it down to 300 °C, and (b) pre-heating the substrate to 700 °C and cooling it down to 500 °C; these cases are called Case 5 and Case 6, respectively.

**Figure 14:**
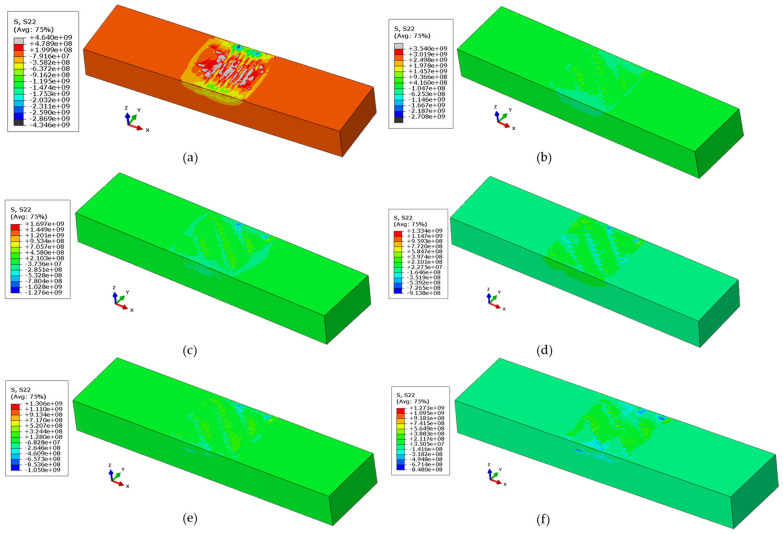
Comparative analysis of longitudinal residual stresses (units in Pa) of (**a**) Case 1, (**b**) Case 2, (**c**) Case 3, (**d**) Case 4, (**e**) Case 5, and (**f**) Case 6.

For all the cases mentioned above, the formation of longitudinal stresses is compared in Figure 14. A continuous decrease in longitudinal stresses can be observed in all cases. In Case 2, pre-heating the substrate to 300 °C resulted in 3500 MPa of tensile stresses, followed by 1697 MPa and 1334 MPa for 500 °C and 700 °C pre-heated substrates, respectively. Interestingly, varying the cooling rate provided even lower tensile stresses, proving to be a much better approach to mitigate residual stresses. Cases 5 and 6, which had a cooling rate from 700 °C to 300 °C and 700 °C to 500 °C, experienced tensile stresses of 1306 MPa and 1271 MPa, respectively. Hence, it can be concluded that higher temperature differences yield more tensile stresses, and vice versa, which has also been supported by the results in [56].

**Figure 15:**
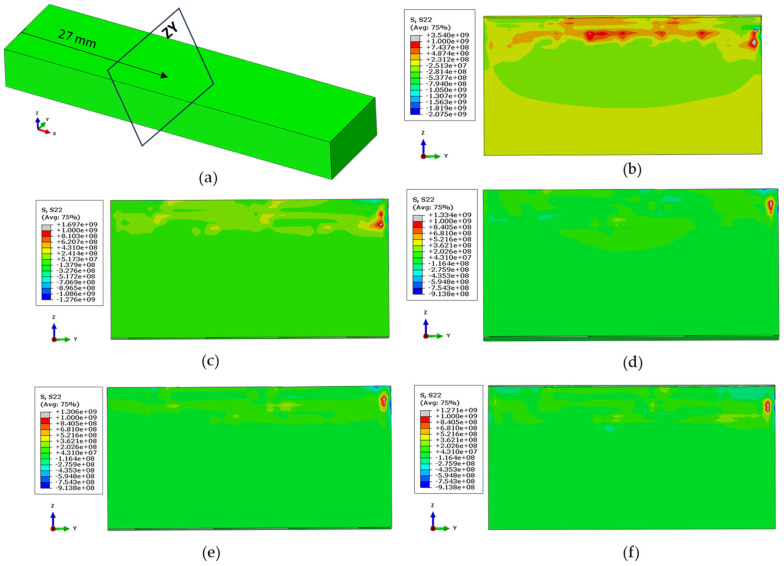
(**a**) Location of section view for longitudinal stresses for (**b**) Case 2, (**c**) Case 3, (**d**) Case 4, (**e**) Case 5, (**f**) and Case 6.

Although Figure 14 discussed the overall distribution of longitudinal stresses, the maximum residuals were not visible at the surface; instead, they were identified within the deposit. Therefore, a section was cut from right to left at 27 mm, revealing the maximum residual stresses and their relative locations from Case 2 to 6, as presented in Figure 15. It may also be noted that for all the cases except Case 1, the location of maximum residual stresses in the length direction was noticed at around 27 mm.

**Figure 16:**
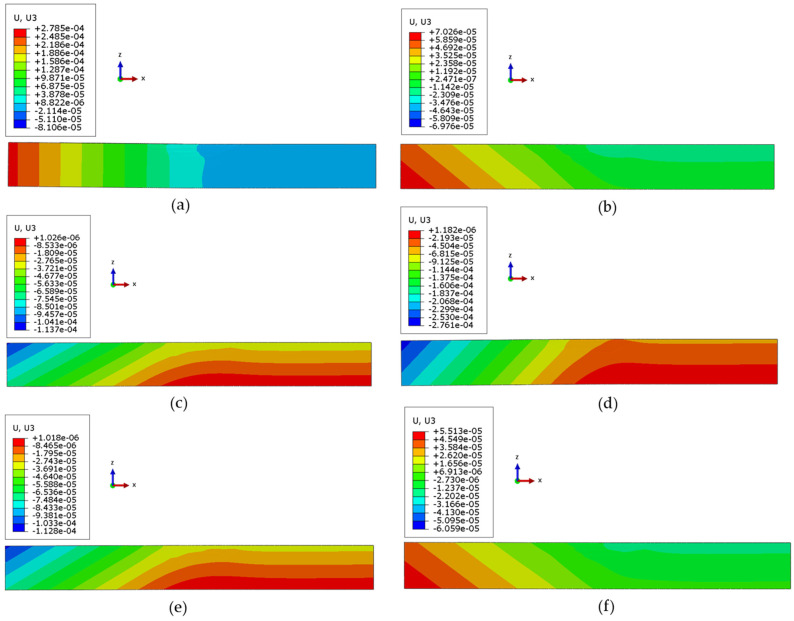
Comparative analysis of final deformation (units in m) for (**a**) Case 1, (**b**) Case 2, (**c**) Case 3, (**d**) Case 4, (**e**) Case 5, and (**f**) Case 6.

A comparison of deformation is presented in Figure 16. It was interesting to notice that in some cases, the repaired part deformed in the opposite direction. Similar results of opposite-direction deformation with various pre-heating effects have also been observed in [49]. The relevant deformations at the final stage, with clamping on one end and showing deformation in the free end, are displayed similarly to Case 1 when compared with previous experiments.

**Figure 17:**
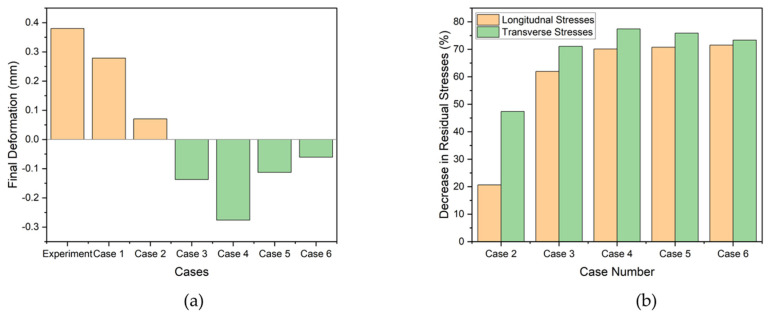
(**a**) Final deformation of all cases and (**b**) percentage decrease in longitudinal and transverse residual stresses.

It was observed that as the pre-heating temperature increases, the deformation decreases until it becomes negative, as shown in Figure 17a. Specific values of 0.0703 mm, −0.137 mm, and −0.276 mm were recorded for Cases 2, 3, and 4, respectively. Conversely, an opposite trend was observed when the cooling rate decreased for Cases 5 to 6. As the cooling rate decreased, the deformation also decreased, with values of −0.113 mm and −0.061 mm for Cases 5 and 6, respectively. Case 6 exhibits the best results, as it demonstrates the minimum deflection in the direction of *Z* or U3, as shown in Figure 17a. Hence, this proves that a lower heat transfer rate after deposition may yield lower deflection.

The quantitative analysis of longitudinal and transverse stresses is presented in Figure 17b. Figure 17b shows the decrease in longitudinal and transverse residual stresses compared to Case 1 or the original simulation with actual boundary conditions. It can be observed that by increasing the pre-heat temperature or decreasing the cooling rate from Case 3 to 6, all the cases exhibit a significant decrease in residual stresses, sometimes by more than 70%, as shown in the graph. If we have to conclude considering both residual stresses and deformation, Case 6 could be a preferred way to follow for parts to be repaired as it shows a significant improvement for both aspects.

## 4. Conclusions

DED, a method within metal additive manufacturing, is well-known for its effectiveness in repairing parts, especially in situations where replacing them is cost-prohibitive. Ensuring that the repaired parts remain free from deformation or residual stresses is essential for preserving their functional integrity. This study performed experimental and numerical analyses on trapezoidal shape repairs, confirming the accuracy of both thermal and mechanical models against experimental data and drawing the following conclusions:

- A sophisticated methodology, utilizing simple trigonometry, was employed to develop a tool path generator capable of accommodating various trapezoidal shapes with differing depths and side wall angles. The tool path is adaptable to process parameters such as scan speed, hatch spacing, and power. This code successfully generates tool paths for both in-house-built systems and event series for Abaqus CAE.
- An uncoupled 3D numerical model was conducted for thermal and mechanical analysis, and its validity was confirmed through comparison with experimental results, including thermal history data and final distortion measurements.
- The study concluded that residual stresses decreased by more than 71% when the substrate was pre-heated to 700 °C and subsequently cooled down to 500 °C (Case 6). Similarly, cooling down to 300 °C (Case 5) also resulted in a reduction of residual stress to 70%.

By implementing a pre-heat and slow-cooling strategy, the final distortion was decreased to a minimum, measuring 0.06 mm, compared to the experimental deformation of 0.38 mm.

## Figures and Tables

**Table 1:** Parameters of Goldak’s double ellipsoid heat source.

*a* (mm)	*b* (mm)	*c_f_* (mm)	*c_r_* (mm)	*f_f_*	*f_r_*
1.1	1.5	2.2	1.1	0.6	1.4

**Table 2:** Ti-6Al-4V temperature dependent material properties.

Temperature (°C)	Density (kg/m^3^)	Thermal Conductivity (W/m. °C)	Heat Capacity (J/kg °C)	Poisson’s Ratio	Thermal Expansion Coefficient (μm/m/°C)	Young’s Modulus (Gpa)	Elastic Limit (Mpa)
20	4420	7	546	0.345	8.78	110	850
205	4395	8.75	584	0.35	10	100	630
500	4350	12.6	651	0.37	11.2	76	470
995	4282	22.7	753	0.43	12.3	15	13
1100	4267	19.3	641	0.43	12.4	5	5
1200	4252	21	660	0.43	12.42	4	1
1600	4198	25.8	732	0.43	12.5	1	0.5
1650	3886	83.5	831	0.43	12.5	0.1	0.1
2000	3818	83.5	831	0.43	12.5	0.01	0.01

## Data Availability

No new data were created or analyzed in this study. Data sharing is not applicable to this article.
